# Risk factors for moderate acute malnutrition among children with acute diarrhoea in India and Tanzania: a secondary analysis of data from a randomized trial

**DOI:** 10.1186/s12887-024-04551-2

**Published:** 2024-01-19

**Authors:** Rodrick Kisenge, Usha Dhingra, Chris A. Rees, Enju Liu, Arup Dutta, Deb Saikat, Pratibha Dhingra, Sarah Somji, Chris Sudfeld, Jon Simon, Per Ashorn, Sunil Sazawal, Christopher P. Duggan, Karim Manji

**Affiliations:** 1 Department of Paediatrics and Child Health, Muhimbili University of Health and Allied Sciences, P.O. Box 65001, Dar Es Salaam, Tanzania; 2 Centre for Public Health Kinetics, New Delhi, India; 3 Division of Paediatric Emergency Medicine, Emory University School of Medicine, Atlanta, USA; 4 Clinical Research Centre, Division of Gastroenterology, Hepatology and Nutrition, Boston Children’s Hospital, Boston, MA USA; 5 Division of Gastroenterology, Hepatology and Nutrition, Boston Children’s Hospital, Boston, MA USA; 6 Harvard T.H. Chan School of Public Health, Boston, USA; 7 The World Health Organization, Geneva, Switzerland

**Keywords:** Moderate Acute Malnutrition, Paediatric, Zinc, India, Tanzania, Acute diarrhoea

## Abstract

**Background:**

Moderate acute malnutrition (MAM) affects over 30 million children aged < 5 years worldwide. MAM may confer a greater risk of developing severe malnutrition and even mortality in children. Assessing risk factors for MAM may allow for earlier recognition of children at risk of deleterious health outcomes.

**Objective:**

To determine risk factors associated with the prevalence and development of MAM among children aged 6 to 59 months with acute diarrhoea who received treatment with oral rehydration solution and zinc supplementation.

**Methods:**

We conducted a secondary analysis of data from a randomized, dose-finding trial of zinc among children with acute diarrhoea in India and Tanzania. We used regression models to assess risk factors for prevalent MAM at the start of diarrhoea treatment and to identify risk factors associated with the development of MAM at 60 days. MAM was defined as weight for length (or height) Z score ≤—2 and > -3 or mid-upper arm circumference < 12.5 and ≥ 11.5 cm.

**Results:**

A total of 4,500 children were enrolled; 593 (13.2%) had MAM at the baseline. MAM at baseline was significantly less common among children in Tanzania than in India (adjusted risk ratio [aRR] 0.37, 95% confidence interval [CI]: 0.30, 0.44, *P* < 0.001), in children aged 24- < 60 months versus 6- < 12 months (aRR 0.46, 95% CI: 0.38, 0.56, *P* < 0.001), and in families with household wealth index higher than the median (aRR 0.79, 95% CI: 0.68, 0.92, *P* = 0.002). Sixty days after outpatient treatment and follow-up, 87 (2.5%) children developed MAM. When compared to children aged 6- < 12 months, children aged 24- < 60 months had a 52% lower risk of developing MAM. Every one unit increase in weight for length (or height) Z score at enrolment was associated with a 93% lower risk of developing MAM during follow-up.

**Conclusions:**

Among children with diarrhoea, younger children and those from households with lower wealth were at greater risk of MAM. These children may benefit from targeted interventions focusing on feeding (targeted nutrition support for at-risk households) and follow up in order to reduce the occurrence of MAM and its consequences.

## Introduction

Undernutrition remains prevalent in low- and middle-income countries. In India 17% of young children are estimated to be wasted while in Tanzania, 5% of children aged ≤ 5 years are wasted [1]. Approximately 40% of the world’s malnourished children live in India [2, 3] and childhood malnutrition contributes to 22% of India’s total disease burden among all age groups [3–6]. Besides, about 34.4% of children below five years in Tanzania are stunted and 14% are underweight [1]. Malnutrition in children is associated with multiple health outcomes, including greater risk of mortality, infections, as well as impaired physical and cognitive development [3–6]. As a result, identifying young children at risk for malnutrition may inform targeted interventions to prevent and treat acute childhood undernutrition.

Moderate acute malnutrition (MAM), defined as weight for length (or height) Z score ≤—2 and > -3 or mid-upper arm circumference < 12.5 and ≥ 11.5 cm, affects over 30 million children aged < 5 years worldwide [7]. Prior studies suggest that MAM affects up to 21% of hospitalized children aged < 5 in India and 5% in Tanzania [7, 8]. MAM confers greater risk for the development of severe acute malnutrition (SAM) and greater risk of childhood mortality [9]. Though much attention has been paid to identifying children at risk of developing SAM in resource-limited settings [10, 11], there is a paucity of studies aimed at determining risk factors for developing MAM.

Assessing risk factors associated with the development of MAM may facilitate early recognition of children at risk of MAM, which may allow for targeted interventions to prevent the development of SAM, mortality, and other sequelae of MAM. Here, our objective was to determine factors associated with the development of MAM among children enrolled in a randomized controlled trial of different doses of zinc for diarrhoea among children in India and Tanzania.

## Methods

### Study design

This secondary analysis was conducted using data from the Zinc Therapeutic Dose Trial (ZTDT) (NCT03078842). ZTDT was an individually randomised, parallel-group, double-blind, controlled trial of three doses of supplemental zinc (5, 10 and 20 mg) conducted among children aged 6–59 months at sites in Delhi, India and Dar es Salaam, Tanzania. Details of the methodology of the trial, and the primary results that showed non-inferiority of lower dose zinc on diarrhoea duration and stool output, have been published elsewhere [12, 13]. Briefly, this trial was conducted from January 2017 to April 2019 and included 4,500 children with diarrhoea lasting < 72 h. After enrolment, children were followed for 60 days with initial close follow up for 15 days to ensure compliance to therapeutic zinc. Subsequently, illnesses were assessed at days 30, 45, and 60. Participant anthropometry was assessed at enrolment (baseline) and 60 days after enrolment.

### Study setting

Participants were recruited from outpatient health facilities in Sangam Vihar, a resettlement colony on the outskirts of South Delhi and Harsh Vihar, a semi-urban locality in North East District, Delhi, India. In Dar es Salaam, Tanzania, recruitment sites were outpatient health facilities in a densely populated peri-urban area (Temeke Municipal Hospital, Mbagala Round Table, and Mbagala Rangi Tatu).

### Anthropometry

Body weight, length/height, and mid-upper arm circumference (MUAC) were measured during screening and 60 days later during a scheduled clinic visit with study staff. Body weight was measured using an electronic baby scale (ADE, ADE GmbH & Co, Hamburg, Germany) or weighing machine (SECA, SECA GmbH & Co, Hamburg, Germany) with reading increment of ± 10 g. If the child was dehydrated, the weight was obtained after rehydration. Length/height was measured by a stadiometer with reading increment of ± 0.1 cm. MUAC was measured in participants’ left upper arm, midway between the olecranon and acromion processes using standard WHO/UNICEF measuring tapes with a reading increment of ± 0.1 cm. Daily calibrations of the anthropometry equipment were conducted to ensure that the equipment produced accurate measurements. Weight measurement tools were calibrated with known weights of 3, 5, 10, and 15 kg. Stadiometers were calibrated using measuring rods of known length measuring 50 cm and 75 cm. Readings of all anthropometric measurements were made in triplicate and the mean of the three readings was used.

### Outcomes and exposures

The primary outcome measure, MAM, was defined as weight-for-height Z score ≤ -2 and > -3 or mid-upper arm circumference < 12.5 and ≥ 11.5 cm according to WHO standards [7]. We performed separate analyses for the presence of MAM at baseline (prevalent MAM) and the development of MAM during follow-up (incident MAM). Assessment of the continuous variable weight-for-length Z scores (WHZ) at baseline and during follow-up was also assessed.

We included variables collected in the parent trial to conduct an exploratory analysis of factors theoretically associated with the development of MAM, both prevalent and incident. In addition, we excluded prevalent MAM and SAM at baseline from analyses of risk factors for incident MAM. Improved water was defined as water from sources like piped water that, by nature of their construction or through active intervention, are protected from outside contamination. Improved sanitation facilities were defined as those that hygienically separate human waste from human contact and include flush or pour-flush [14]. Dysentery was defined as acute diarrhoea with blood or mucous in stool. Household wealth index was defined as a measure of a household’s standard of living. A country-specific household wealth index was constructed by means of a principal components analysis of these factors: household ownership, household assets, drinking water source, and sanitation.

### Statistical analysis

Descriptive statistics were used to summarize baseline characteristics of caregivers and children who participated in the trial. Frequencies were presented for categorical variables and mean ± standard deviation (SD) for continuous variables. Univariable log-binomial regression analyses were used to estimate risk ratios (RRs) and 95% confidence intervals (CIs) for predictors of the outcome of interest to produce relative risks. Variables significant at the *P* < 0.20 were retained in a multivariable log-binomial regression analysis model to calculate adjusted RRs. *P* values < 0.05 were considered statistically significant. Analyses were performed using SAS software Version 9.3 (SAS Institute, Cary, NC, USA).

## Results

There were 4,500 children enrolled in the trial (2,250 at each site). The mean age of enrolled children was 5.0 (± 26.8) years. A total of 593 (13.2%) children had prevalent MAM, 47 (1%) children had severe acute malnutrition (SAM), and 1,188 (26.4%) children were stunted at enrolment (Table 1).

**Table 1:** Baseline Characteristics of 4500 children and caretakers enrolled in a zinc dosing trial for acute diarrhoea

	n (%) or mean (± SD)
**Study site,** n (%)	
India	2250 (50.0)
Tanzania	2250 (50.0)
**Maternal Characteristics**	
Age (years)	5.0 (± 26.8)
Education (years)	4.1 (± 7.2)
Education > 7 years, n (%)	1990 (44.8)
**Household Characteristics**	
Household wealth index above median, n (%)	2270 (50.5)
Improved water, n (%)	4455 (99.2)
Improved sanitation, n (%)	4463 (99.4)
**Child Characteristics**	
Age in months at randomization	14.9 (± 23.0)
Age group at randomization	
6 < 12 months, n (%)	1256 (27.9)
12 to < 24 months, n (%)	1477 (32.8)
24 to < 60 months, n (%)	1767 (39.3)
Male, n (%)	2345 (52.1)
Breastfeeding on day before enrolment, n (%)	2586 (57.6)
Rotavirus vaccination, n (%)	2238 (49.7)
Duration of diarrhoea before enrolment	
≤ 24 h, n (%)	165 (3.7)
25 to 48 h, n (%)	3714 (82.5)
49 to < 72 h, n (%)	621 (13.8)
Number of loose or watery stools the child passed in previous 24 h	5.7 (± 2.1)
Dysentery, n (%)	167 (3.7)
Some dehydration, n (%)	56 (1.2)
Axillary temperature > 38 °C, n (%)	122 (2.7)
Observed respiratory rate > 40 breaths/min, n (%)	255 (5.7)
Cough or difficulty breathing, n (%)	1267 (28.2)
Previous use of antibiotics, n (%)	96 (2.1)
Length/height-for-age z-score	1.2 (± -1.3)
Weight-for-age z-score	1.1 (± -1.2)
Weight-for-length/height z-score	1.0 (± -0.7)
Mid–upper-arm circumference for age z score	1.0 (± -0.8)
Underweight at enrolment, n (%)	1034 (23.0)
Stunted at enrolment, n (%)	1188 (26.4)
Wasted at enrolment run, n (%)	407 (9.0)
Malnutrition status at enrolment	
Severe acute malnutrition, n (%)	47 (1.0)
Moderate acute malnutrition, n (%)	593 (13.2)
Not malnourished, n (%)	3860 (85.8)
Plasma zinc concentration at enrolment — µg/dl	73.5 (24.9)
Plasma zinc concentration at enrolment < 65 µg/dl, n (%)	492 (37.6)

Severe acute malnutrition was defined as WHZ/WLZ <  − 3 or MUAC < 11.5 cm Moderate acute malnutrition was defined as WHZ/WLZ ≥  − 3 and <  − 2 or MUAC ≥ 11.5 cm and < 12.5 cm

At baseline and adjusting for the following variables; study site, maternal age > 25, child age in months, household wealth index above median, duration of diarrhoea before enrolment, observed respiratory rate > 40 breaths/min, cough or difficulty breathing and plasma zinc concentration at enrolment < 65 µg/dl, MAM was significantly less common among children in Tanzania than those in India (adjusted risk ratio [aRR] 0.37, 95% CI: 0.30, 0.44, *P* < 0.001) (Table 2). Compared with children 6– < 12 months of age, children ages 12– < 24 months (aRR 0.83, 95% CI: 0.70, 0.99, *P* < 0.04) and children aged ≥ 2 years (aRR 0.46, 95% CI: 0.38, 0.56, *P* < 0.001) had a significantly lower risk of prevalent MAM. Children who lived in a household with a wealth index above the median (aRR 0.79, 95% CI: 0.68, 0.92, *P* = 0.002) also had lower risk of prevalent MAM.

**Table 2:** Relative Risks for Prevalent MAM by Baseline Participant Characteristics (*n* = 4453)

	Univariate		Multivariate	
Variables	Prevalence Ratio	*P* value	Prevalence Ratio	*P* value
**Country**				
India	Ref		Ref	
Tanzania	0.41 (0.35, 0.49)	** < 0.001**	0.37 (0.30, 0.44)	< 0.001
**Maternal age > 25 years**				
No	Ref		Ref	
Yes	0.82 (0.70, 0.95)	**0.008**	0.92 (0.80, 1.07)	0.30
**Maternal education > 7 years**				
No	Ref			
Yes	0.94 (0.80, 1.09)	0.39		
**Child age, month**				
6- < 12	Ref		Ref	
12 to < 24	0.85 (0.71, 1.02	**0.07**	0.83 (0.70, 0.99)	**0.04**
24 to < 60	0.57 (0.48, 0.69)	** < 0.001**	0.46 (0.38, 0.56)	** < 0.001**
**Child sex**				
Female	Ref			
Male	0.96 (0.83, 1.12)	0.61		
**Household wealth index above median**				
No	Ref		Ref	
Yes	0.78 (0.67, 0.91)	**0.002**	0.79 (0.68, 0.92)	**0.002**
**Breast-feeding before enrolment**				
No	Ref			
Yes	1.33 (1.14, 1.55)	** < 0.001**		
**Duration of diarrhoea before enrolment**				
< = 24 h	Ref		Ref	
25 to 48 h	0.86 (0.60, 1.23)	**0.40**	0.79 (0.55, 1.13)	0.20
49 to < 72 h	0.67 (0.44, 1.02)	**0.06**	0.93 (0.60, 1.42)	0.72
**Dysentery**				
No	Ref			
Yes	0.81 (0.52, 1.26)	0.35		
**Dehydration**				
No	Ref			
Yes	1.35 (0.76, 2.37)	0.30		
**Axillary temperature > 38 °C**				
No	Ref			
Yes	0.86 (0.52, 1.41)	0.55		
**Observed respiratory rate > 40 breaths/min**				
No	Ref		Ref	
Yes	0.61 (0.41, 0.93)	**0.02**	0.72 (0.47, 1.10)	0.13
**Cough or difficulty breathing**				
No	Ref		Ref	
Yes	1.29 (1.10, 1.51)	**0.001**	1.04 (0.89, 1.22)	0.60
**Previous use of antibiotic agent**				
No	Ref			
Yes	1.26 (0.80, 1.98)	0.32		
**Plasma zinc concentration at enrolment < 65 µg/dl**				
No	Ref		Ref	
Yes	0.79 (0.59, 1.04)	**0.09**	1.13 (0.86, 1.49)	0.38
**Improved water**				
No	Ref			
Yes	0.66 (0.34, 1.29)	0.23		
Improved sanitation				
No	Ref			
Yes	1.24 (0.43, 3.64)	0.69		

MAM was defined as WHZ/WLZ ≥  − 3 and <  − 2 or MUAC ≥ 11.5 cm and < 12.5 cm

Children with SAM (WHZ < -3 or MUAC < 11.5 cm) were excluded from the analysis

Variables with *P* < 0.10 in univariate analysis were included in multivariate analysis. These variables were study site, maternal age > 25, child age in months, household wealth index above median, duration of diarrhoea before enrolment, observed respiratory rate > 40 breaths/min, cough or difficulty breathing and plasma zinc concentration at enrolment < 65 µg/dl

For the analysis of incident MAM, after excluding children who were lost to follow-up or developed SAM, 3,537 children were included in the analysis (Table 3). Of these, 87 (2.5%) developed MAM during follow-up. Despite excluding prevalent cases of MAM and SAM at baseline from analyses of risk factors for incident MAM, when compared to children in India, those in Tanzania had a 33% lower risk of developing incident MAM during the 60-day follow-up (*P* = 0.06). When compared to children aged 6- < 12 months, those aged 24- < 60 months old had a 52% lower risk (aRR 0.48, 95% CI: 0.27, 0.85, *P* < 0.01) of developing incident MAM. Moreover, every one unit increase in weight for length (or height) Z score at enrolment was associated with a 93% lower risk (aRR 0.07 95% CI: 0.04, 0.11, *P* < 0.001) of developing incident MAM. Further, in unadjusted analyses, maternal education, child sex, dysentery, fever, prior antibiotic use, improved water or improved sanitation were not associated with the development of moderate acute malnutrition.

**Table 3:** Relative Risks for Incident MAM by Participant Characteristics (*n* = 3535)

	Univariate		Multivariate	
Variables	Risk Ratio	*P* value	Risk Ratio	*P* value
**Country**				
India	Ref		Ref	
Tanzania	0.62 (0.40, 0.94)	**0.02**	0.67 (0.45, 1.01)	**0.06**
**Maternal age > 25 years**				
No	Ref			
Yes	1.14 (0.75, 1.73))	0.55		
**Maternal education > 7 years**				
No	Ref			
Yes	0.99 (0.65, 1.50	0.95		
**Child age, month**				
6- < 12	Ref		Ref	
12 to < 24	1.86 (1.07, 3.21)	**0.03**	1.24 (0.74, 2.07)	0.41
24 to < 60	0.97 (0.54, 1.74)	**0.92**	0.48 (0.27, 0.85)	**0.01**
**Child sex**				
Female	Ref			
Male	1.01 (0.66, 1.53)	0.97		
**Breast-feeding before enrolment**				
No	Ref			
Yes	1.29 (0.84, 1.98)	0.24		
**Household wealth index above median**				
No	Ref			
Yes	1.02 (0.67, 1.55)	0.92		
**Duration of diarrhoea before enrolment**				
< = 24 h	Ref			
25 to 48 h	0.79 (0.29, 2.13)	0.64		
49 to < 72 h	0.71 (0.23, 2.19)	0.55		
**Dysentery**				
No	Ref			
Yes	0.89 (0.28, 2.78)	0.84		
**Dehydration**				
No	Ref			
Yes	1.04 (0.15, 7.30)	0.97		
**Axillary temperature > 38 °C**				
No	Ref			
Yes	1.62 (0.61, 4.34)	0.33		
**Observed respiratory rate > 40 breaths/min**				
No	Ref			
Yes	0.77 (0.28, 2.07)	0.60		
**Cough or difficulty breathing**				
No	Ref			
Yes	1.00 (0.63, 1.59)	1.00		
**Previous use of antibiotic agent**				
No	Ref			
Yes	Non-estimable due to zero			
**Plasma zinc concentration at enrolment < 65 µg/dl**				
No	Ref			
Yes	0.65 (0.27, 1.56)	0.34		
**Weight-for-Height z-score at enrolment**	0.07 (0.04, 0.11)	** < 0.001**	0.07 (0.04, 0.11)	** < 0.001**
**Duration of episode, days**				
< 3	Ref			
3–5	1.04 (0.67, 1.60)	0.87		
> 5	0.88 (0.35, 2.19)	0.79		
Number of loose or watery stools after enrolment*				
< 7	Ref			
7–9	1.10 (0.57, 2.14)	0.78		
10–12	0.95 (0.55, 1.65)	0.86		
> 12	0.83 (0.48, 1.40)	0.48		
**Dose of Zinc intervention**				
20 mg	Ref			
10 mg	1.31 (0.81, 2.12)	0.27		
5 mg	0.73 (0.42, 1.28)	0.27		
**Improved water**				
No	Ref			
Yes	0.64 (0.09, 4.40)	0.62		

MAM was defined as WHZ/WLZ ≥  − 3 and <  − 2 or MUAC ≥ 11.5 cm and < 12.5 cm

Children with MAM (*n* = 593) or SAM (*n* = 47) at enrolment and those without WHZ and MUAC (*n* = 323) measures or developed SAM (*n* = 2) during the study period were excluded from this analysis

We excluded prevalent cases of MAM and SAM at baseline from analyses of risk factors for incident MAM

Variables with *P* < 0.10 in univariate analysis were included in multivariate analysis. These variables were study site, child age in months and weight-for-Height z-score at enrolment)

## Discussion

In this secondary analysis of a randomized trial including 4,500 children at sites in India and Tanzania, MAM was common at enrolment. Among children with diarrhoea, younger children, those living in India, and those from households with less wealth were at greater risk of prevalent MAM. The high rate of prevalent a MAM in this study highlights the fact that insufficient nutritional status in children aged < 5 years in Tanzania and India may be an important risk factor for diarrhoea. Greater fluid losses through diarrhoea and reduced oral intake during these illnesses may contribute to worsening nutritional status [13–15]. At enrolment, MAM was less common among children at the site in Tanzania compared to children in India, which may be indicative of differences in study locations, populations, and underlying health and nutritional factors.

Household wealth played an important role in lowering the risk of prevalent MAM in our study, which aligns with results from previous studies conducted in low- and middle-income countries that demonstrated weight faltering or poor weight gain following an acute illness due to an infection, such as diarrhoeal disease [16–19]. Though not measured in this study, the built environment in which a child lives is crucial to determining their access to food and the diversity of foods available for their consumption [4, 9, 16]. Younger age and lower household wealth are key indicators of the development of MAM and these populations should be targeted in future therapeutic feeding programs. Additionally, higher WHZ at enrolment was associated with a lower risk of incident MAM during follow-up, which indicates the importance of good nutritional status in the prevention of developing MAM during an acute illness like diarrhoea in children.

We found that a number of factors that previous studies [5–7] have identified as risk factors for both prevalent and incident MAM (e.g., maternal education, dehydration, low plasma zinc and child sex) were not associated with MAM in our analyses. This might have been due to the strong association with study site and other identified risk factors, the absence of a biologic relationship, or limited power in the case of the relatively rare event of incident MAM.

### Limitations

This study’s results should be interpreted in the context of several limitations. Since this was not a planned secondary analysis, the original trial was not powered a priori to primarily detect changes in anthropometry among enrolled children. Additionally, there are other potentially unmeasured confounders that were not accounted for in this study. Specifically, we did not measure dietary intake or the use of ready to use supplemental feeds, which may have prevented the development in MAM in some children. However, ready to use supplemental feeds are infrequently administered to children without malnutrition or with MAM in low- and middle-income countries including India and Tanzania [7, 15]. Furthermore, local ecology or geographical environment, residential culture, maternal knowledge of diarrhoea treatment, and government policies combating malnutrition and diarrhoea which are factors that may have a significant impact on development of MAM were not collected in our study and thus were not factored into our regression model. Finally, the incidence of MAM after enrolment (2.5%) was very rare, making our analyses of incident MAM likely underpowered.

## Conclusions

Among children with diarrhoea, younger children and those from households with less wealth were at greater risk of MAM. These children should be prioritized in interventions that target feeding and follow up among children with diarrheal illnesses to reduce the occurrence of MAM as well as its sequelae and to contribute towards achieving Sustainable Development Goal number two (SDG 2) and Global Nutritional targets that aims at tackling of undernutrition and hunger. In addition, improving the "stock of health" (proxied by nutritional status at baseline) can be an effective strategy for preventing more severe illness when episodes of childhood diarrheal disease occur in low-resource environments.

## Data Availability

The datasets analysed during this current study are available on reasonable request to kpmanji@gmail.com.

## Abbreviations

CI: Confidence Interval
MAM: Moderate Acute Malnutrition
MUAC: Mid Upper Arm Circumference
SAM: Severe Acute Malnutrition
UNICEF: The United Nations Children’s Fund
WAZ: Weight for Age Z score
WHO: World Health Organisation
ZTDT: Zinc Therapeutic Dose Trial
